# Combining Synthetic Human Odours and Low-Cost Electrocuting Grids to Attract and Kill Outdoor-Biting Mosquitoes: Field and Semi-Field Evaluation of an Improved Mosquito Landing Box

**DOI:** 10.1371/journal.pone.0145653

**Published:** 2016-01-20

**Authors:** Nancy S. Matowo, Lizette L. Koekemoer, Sarah J. Moore, Arnold S. Mmbando, Salum A. Mapua, Maureen Coetzee, Fredros O. Okumu

**Affiliations:** 1 Environmental and Ecological Sciences Thematic Group, Ifakara Health Institute, Dar es Salaam, Tanzania; 2 Wits Research Institute for Malaria, School of Pathology, Faculty of Health Sciences, University of the Witwatersrand, Johannesburg, South Africa; 3 Centre for Opportunistic, Tropical and Hospital Infections, National Institute for Communicable Diseases, Johannesburg, South Africa; 4 Swiss Tropical and Public Health Institute, Basel, Switzerland; 5 University of Basel, Basel, Switzerland; 6 School of Public Health, Faculty of Health Sciences, University of the Witwatersrand, Johannesburg, South Africa; University of Tours, FRANCE

## Abstract

**Background:**

On-going malaria transmission is increasingly mediated by outdoor-biting vectors, especially where indoor insecticidal interventions such as long-lasting insecticide treated nets (LLINs) are widespread. Often, the vectors are also physiologically resistant to insecticides, presenting major obstacles for elimination. We tested a combination of electrocuting grids with synthetic odours as an alternative killing mechanism against outdoor-biting mosquitoes.

**Methods:**

An odour-baited device, the Mosquito Landing Box (MLB), was improved by fitting it with low-cost electrocuting grids to instantly kill mosquitoes attracted to the odour lure, and automated photo switch to activate attractant-dispensing and mosquito-killing systems between dusk and dawn. MLBs fitted with one, two or three electrocuting grids were compared outdoors in a malaria endemic village in Tanzania, where vectors had lost susceptibility to pyrethroids. MLBs with three grids were also tested in a large semi-field cage (9.6×9.6×4.5m), to assess effects on biting-densities of laboratory-reared *Anopheles arabiensis* on volunteers sitting near MLBs.

**Results:**

Significantly more mosquitoes were killed when MLBs had two or three grids, than one grid in wet and dry seasons (P<0.05). The MLBs were highly efficient against Mansonia species and malaria vector, *An*. *arabiensis*. Of all mosquitoes, 99% were non-blood fed, suggesting host-seeking status. In the semi-field, the MLBs reduced mean number of malaria mosquitoes attempting to bite humans fourfold.

**Conclusion:**

The improved odour-baited MLBs effectively kill outdoor-biting malaria vector mosquitoes that are behaviourally and physiologically resistant to insecticidal interventions e.g. LLINs. The MLBs reduce human-biting vector densities even when used close to humans, and are insecticide-free, hence potentially antiresistance. The devices could either be used as surveillance tools or complementary mosquito control interventions to accelerate malaria elimination where outdoor transmission is significant.

## Background

Current interventions against malaria vectors, notably long-lasting insecticidal nets (LLINs) and indoor residual spraying (IRS), have been highly successful against the disease [1]. There has also been a decrease in malaria mortality rates between 2000 and 2013 by 47% worldwide and by 54% in Africa, where about 9 in 10 of the malaria deaths occur, mainly in children below five years old [2]. However, malaria remains endemic in sub-Saharan Africa, with an estimate of 198 million malaria cases and 584,000 deaths annually around the world, according to the latest World Malaria Report [2] by the World Health Organization (WHO). Despite the obvious successes and optimism expressed in current strategies, evidence suggests that in order to achieve the target of malaria elimination as stated in the newly launched Global Technical Strategy for Malaria (GTSM), 2016–2030, there are still numerous challenges that must be addressed [3].

One of these challenges is the fact that, while LLINS and IRS have effectively controlled mosquitoes that bite humans indoors and rest indoors [4], a significant proportion of malaria transmission is thought to still happen outdoors [5,6]. Nevertheless, there are other vector species that rest and bites humans or other vertebrates, outdoors [7,8] and are for that reason it becomes hard to control using only the indoor interventions [9]. This outdoor transmission is likely mediated mostly by mosquitoes that naturally bite outdoors and those that have developed behavioural resistance (e.g., avoidance of contact with lethal insecticidal surfaces and/or change of their biting time), adaptive behaviour such as biting people when outdoors [10] or physiological resistance (i.e., failure to be killed after adequate contact with otherwise toxic doses of insecticides) [5].

Various technologies have been proposed for outdoor-mosquito control, one of which is the use of devices baited with synthetic human odours to lure, trap and kill malaria vectors [11,12].

Lure and kill technology has great potential for use in Dengue control mediated by daytime biting *Aedes aegypti* mosquitoes that cannot be controlled by LLINs. Good efficacy has been demonstrated using oviposition traps baited with volatile grass mixtures as a synthetic oviposition lures for gravid female *Ae*. *aegypti* [13,14] and mass trapping of host seeking female *Ae*. *aegypti* using odour-baited Biogent-Sentinel (BG-S) traps [15,16]. In addition, lure and kill technology has been extremely successful against arthropods with low reproductive output such tsetse fly vectors of African trypanosomiasis [17] and have theoretical potential against malaria transmission [18]. There is however, only one large scale study currently underway to demonstrate their effectiveness against malarial mosquitoes [19]. Thus, there is a need to further evaluate and refine the technology before it can be recommended for large-scale use.

Recently, an outdoor mosquito control device, named the odour-baited mosquito landing box (MLB), was developed to attract and kill outdoor host-seeking mosquitoes, including major malaria vectors *An*. *arabiensis* and *An*. *funestus* s.s [12]. During the initial tests, the mosquitoes attracted to the MLB spent only brief periods of time around the device [12], thus limiting lethal contact even when the MLBs were covered with paint-based mixtures of highly effective organophosphates such as pirimiphos-methyl emulsified concentrate. We hypothesised that the reasons for this included the avoidance responses of *An*. *arabiensis*, and other mosquito species on conventionally treated insecticidal surfaces that have contact irritancy or non-contact excito-repellent effects [20].

Another possibility was that naturally, the mosquitoes were attracted to the lure but gave up and left the MLB after they found no short-range host cues. Similar behaviours have been observed in experimental hut trials with un-holed LLINs where mosquitoes enter houses to feed but when they are unable to feed on a host escape promptly before adequate contact with sub-lethal doses of either pyrethroids or organochloride insecticides [21]. Mosquitoes also leave experimental huts earlier in the presence of irritant insecticides such as pyrethroids [20,22,23]. However, this behaviour was less pronounced when organophosphates were used in impregnated nets but only after washing [24]. In situations where vector species such as *An*. *arabiensis* naturally have alternative blood hosts, e.g., cattle [25,26], and where such vectors contribute significantly to ongoing malaria transmission outdoors, it is important to identify means of killing the vectors instantly when they first make contact with the MLB. In addition, physiological resistance in malaria vector populations is rapidly spreading across Africa and is likely to be a major obstacle to ongoing vector control efforts [27] thus the need to explore non-insecticidal means of killing mosquitoes.

Electrocuting grids (EGs) have been used widely in studying vector behaviours such as flight, oviposition and host seeking responses of both tsetse fly and mosquitoes [28–31]. The technique also has been deployed at the household level and in commercial areas such as restaurants and bars for trapping and killing houseflies and other nuisance flying insects that are visually attracted to light [32,33]. However, such commercially available EGs are not regularly used outdoors in local rural settings [34] because they are expensive and require electrical power. Another limitation of these EG devices is that most of the existing versions are not sufficiently robust under natural settings and not simple to construct locally, especially in rural communities in malaria-prone areas [34].

**Fig 1:**
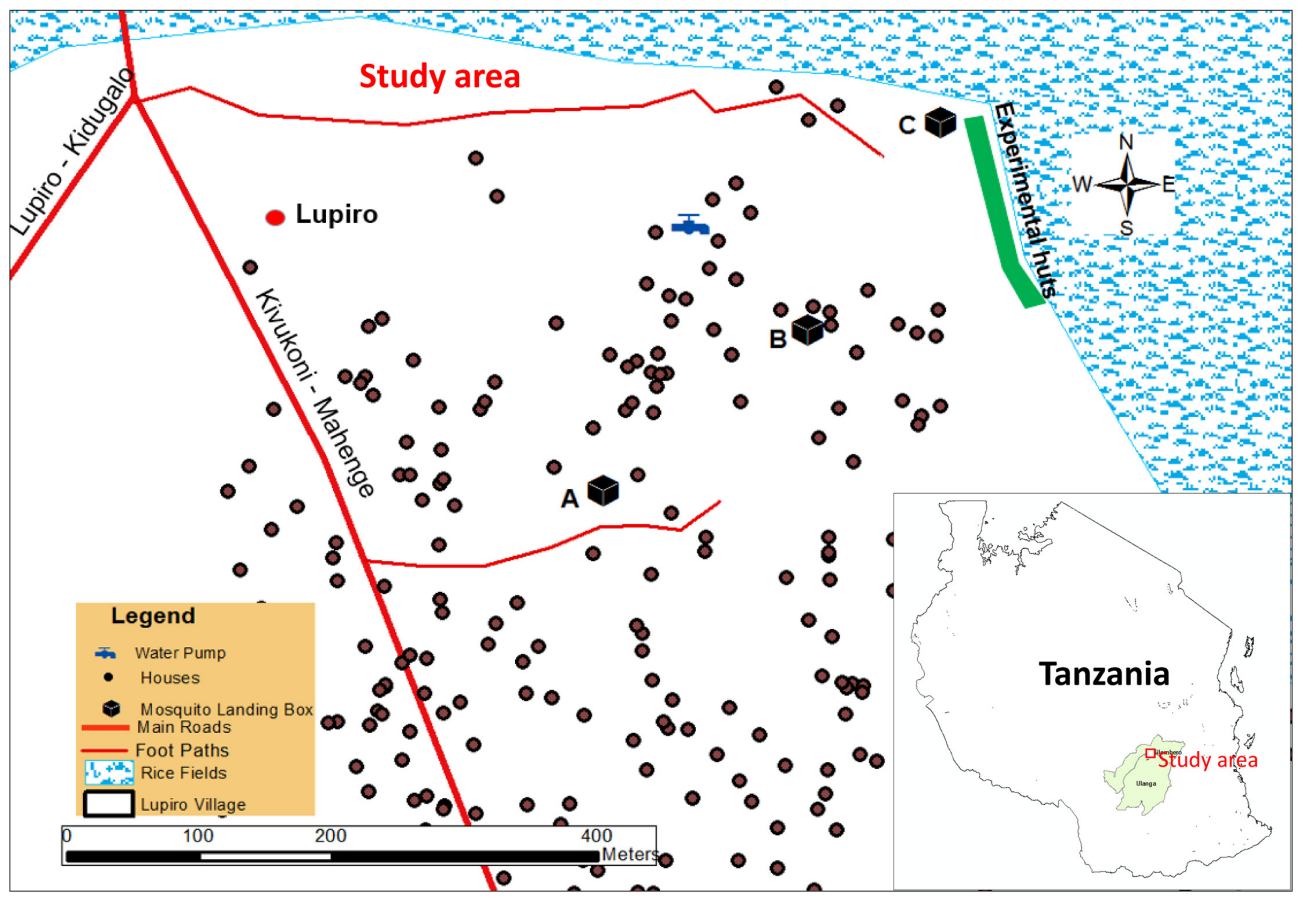
Map showing geographical positioning of the MLBs at A, B and C in Lupiro village, south-eastern Tanzania (Courtesy of Alex J. Limwagu).

Here we report improvements to the MLB to address these challenges for improved efficiency, to conserve energy, and to minimize the need for human handling. In summary, these modifications (Fig 1) included: 1) fitting low-cost electrocuting grids (EGs) onto the sides of the MLB to instantly kill transient host-seeking mosquitoes; and 2) the addition of an all-weather light-sensor which automatically switched both the electrocuting system and the odour-dispensing system on at dusk and off at dawn, thus improving safety, saving energy and reducing human handling. The MLB [12] fitted with low-cost, commercially available grids would instantly kill host-seeking mosquito populations effectively where mortality from insecticide treated surfaces is lower due to behavioural avoidance characteristics [7,10,20] or physiological resistance [5,6]. The simple type of electrocuting grid that we used here is widely used domestically for killing flying insects including mosquitoes just by swatting the flies mid-air. The grids fitted in the improved MLB are powered by the solar panel that powers the odour-dispensing unit.

This work was motivated by the need for an effective odour-baited mosquito control device that is low-cost, environmentally friendly, non-hazardous to people, easy to maintain, robust and efficient against target vectors.

## Methods

### Study area

The field study was carried out in Lupiro village (8.385° S, 36.670° E), in the Ulanga district of south-eastern Tanzania, an area of approximately 300 meters above sea level, at the edge of the Kilombero river flood plain (Fig 1). The MLBs with one, two or three grids were positioned at three different locations mainly position A, B and C (Fig 1). The study area experiences two main climatic seasons, a wet season, which peaks between March and June, and the dry season, which peaks between August and October. Annual rainfall is 1200–1800 mm, and the mean daily temperatures are 20–32°C. Most houses in the area are constructed with clay brick walls with either iron or grass-thatched roofs, but a significant proportion have openings on windows, doors and eave spaces. The predominant malaria vectors are the *An*. *arabiensis* and *An*. *funestus* groups. Interestingly, *Anopheles gambiae s*.*s*., which historically dominated the area, is now rarely found, mainly as a result of extensive use of bed nets [4].

Recently, a report of standard WHO insecticide susceptibility tests [35] on field collected adult mosquitoes from this village, showed that *An*. *arabiensis* were 100% susceptible to dichlorodiphenyltrichloroethane (DDT) but highly resistant to all pyrethroids [36]. Over the past decade, the Kilombero valley, which was hyper-endemic in the early 2000s, has experienced more than 50% reduction in malaria prevalence and is now classified as meso-endemic [37]. Outdoor transmission is rising and has been claimed to contribute significant proportions of the residual malaria transmissions [5,38].

The semi-field study was conducted at the Ifakara Health Institute’s experimental station located in Kining’ina village (8.11417° S, 36.67864° E) in Kilombero district, south-eastern Tanzania, approximately 6km north of Ifakara town. These experiments were conducted inside two chambers of a large semi-field system (SFS), i.e. screen house chambers measuring (9.6m × 9.6m × 4.5m), as described in [39–41]. During the experiments, the average daily temperature and relative humidity inside the SFS varied between 20.5°C—29.0°C and 50.5% - 80.0%, respectively (The experimental design is described below).

### Improvement of the Mosquito Landing Boxes by fitting low-cost electrocuting grids and light sensors that trigger automatic on/off operation

**Fig 2:**
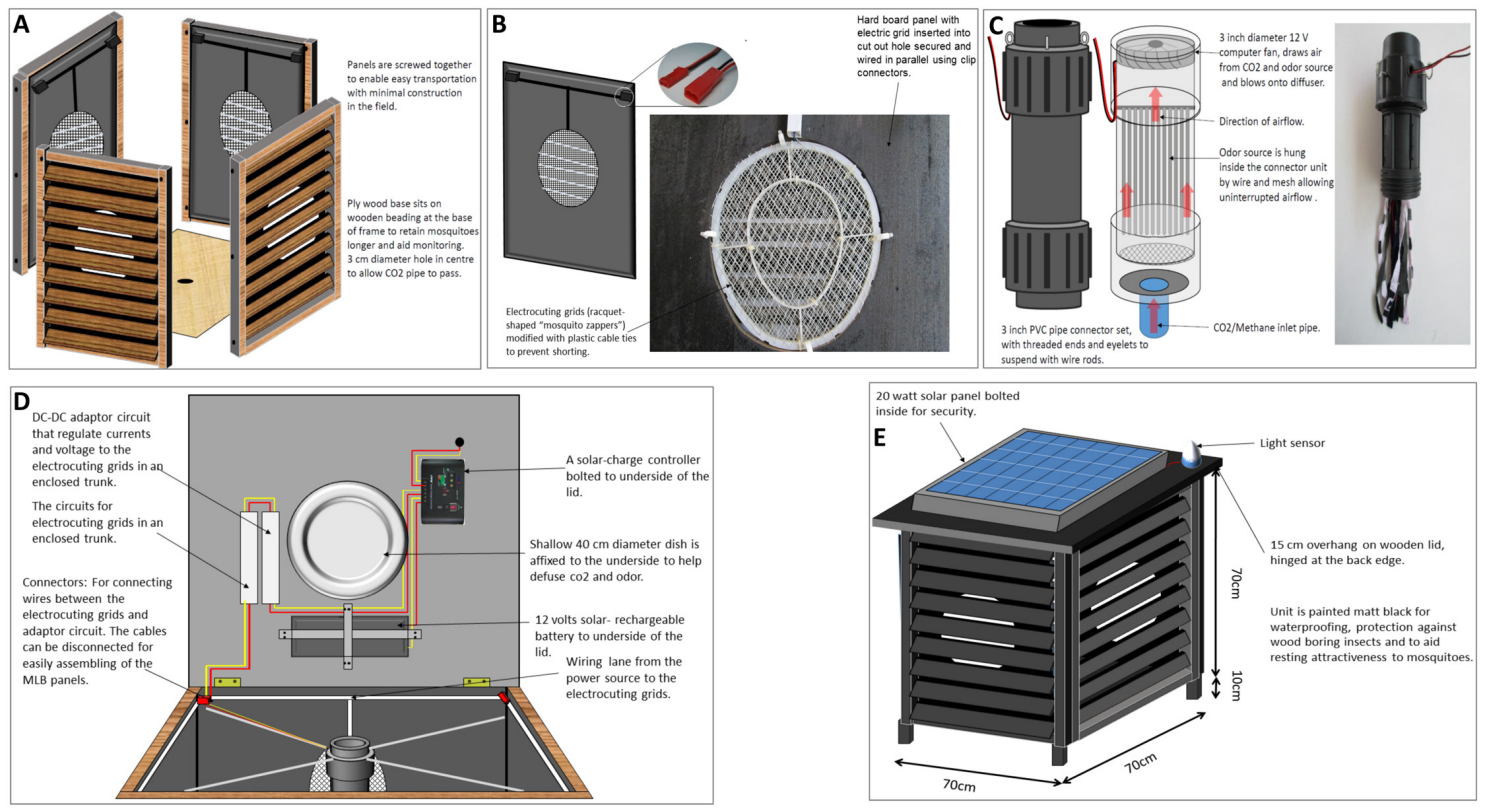
(A) The panels of the Odour-baited Mosquito Landing Box (MLB), fitted with low-voltage electrocuting grids (EGs). (B) A close look of the EG inserted and secured into a hard wooded board of the panel of the MLB. (C) The odour-dispensing unit of the MLB. (D) Both the electrical system and the odour-dispensing unit are powered by a 12-volts solar-rechargeable battery regulated by solar-charger controller and adapter circuit system, allowing for a passive but effective mosquito control and surveillance system. (E) An automated on/off light sensitive switch that activates the device at dusk and stops it at dawn.

To improve efficiency and ease of use, the MLBs were fitted with solar-driven low-voltage electrocuting grids (EGs) to kill attracted mosquitoes instantly without destroying essential morphological and molecular features for mosquito identification and pathogen monitoring (Fig 2A and 2B).

Commercially available racquet-shaped “mosquito zappers” (manufactured by *LiTian Electronic Limited Company*, *Yiwu City*, *China*), were purchased at the equivalent of 4 USD/unit from local retail stores and modified (by inserting plastic cable between the grid wires to prevent short-circuiting) for use as the grids on the sides of the MLB (Fig 2B). The grids were supported by rectangular pieces of hard board on each side of the box so that only a small circular portion of the soft boards, equal to the size of the grids, were visibly open whenever the landing surfaces were tilted (Fig 2B). The EGs were inserted on the inside sections behind the louvers and powered by the solar system which ran the odour-dispensing system. The louvers were adjusted to an angle to allow attracted mosquitoes to pass through, but also provide protection of the grids against rainfall. The energy that drives the 12 volts fan of the odour-dispensing system of the MLB (Fig 2C) from the solar-rechargeable battery was only regulated by the solar charger-controller (Fig 2D). To be able to minimize excess currents that could burn and destroy the morphological features of the mosquitoes, the electricity coming from the solar battery through the charger controller was regulated by DC-DC adapter circuit with an output range of 1.5–3 volts and a maximum 1.2 amperes and supplied to the EGs (Fig 2D). Due to some voltage drop, the voltages and currents measured in the EGs were between 0.28 and 0.75 volts and 0.35 and 1.39 amperes, respectively. We also fitted an all-weather-light sensitive sensor to switch on the odour-dispensing unit and the grids of the MLB at dusk and switch them off at dawn (Fig 2E). This automated system ensured that: *a)* the solar battery power is saved and unused during the day, *b)* the devices are not in any way hazardous to children who might touch them during the day, and *c)* no daily human servicing is necessary for these devices, other than the experimenter collecting data.

The estimations of potential features including community acceptance, sustainability, communal benefits, limitations and target-product profile of the effective odour-baited mosquito traps has been described in detail in the previous article [18]. The original cost of fabrication, purchasing of electrical solar parts, assembling, and annual maintenance of one experimental MLB prototype was 190 USD. However, the cost will be lower following mass production of the MLBs using cheaper and long-lasting materials, preferably plastic which is estimated to be 25USD per MLB (personal communication with Sarah J. Moore), and or adopting a community-based delivery mechanism.

### Comparative evaluation of the killing efficacy of MLBs fitted with electrocuting grids against wild populations of free-flying mosquitoes

Three MLBs (the first one fitted with an EG on only one side, the second fitted with EGs on two sides, and the third fitted with EGs on three sides) were positioned 50–150 meters apart, and at least 30 meters from the nearest house, in the study village (Fig 1). White linoleum was placed at the bottom of each of the devices so that any dead mosquitoes dropping down could be recovered and counted. The boxes were raised on four pedestals in water moats to prevent any ants from entering and scavenging the dead mosquitoes.

The MLBs were baited with the Ifakara blend of mosquito attractants [42], a highly attractive synthetic human odour that was demonstrated in experimental hut studies to be more attractive than humans at long-range. The blend comprises hydrous solutions of ammonia (2.5%), L-lactic acid (85%), and other aliphatic carboxylic acids, namely propionic acid (C3) at 0.1%, butanoic acid (C4) at 1%, pentanoic acid (C5) at 0.01%, 3-methylbutanoic acid (3mC4) at 0.001%, heptanoic acid (C7) at 0.01%, octanoic acid (C8) at 0.01% and tetradecanoic acid (C14) at 0.01% dispensed via nylon strips, supplemented with CO_2_ gas flowing at 500 ml/min [12,42,43]. The odour-dispensing system was similar to the one described in [12], and was powered by an automated solar-battery driven mechanism (Fig 2C).

The MLBs with one, two or three grids were compared using a 3 × 3 fully randomised Latin square experiment replicated over 21 nights, in three different locations in the study village (Fig 1):

Location A was inside the village, approximately 150 m from the edge of the village, and the device was located 30 m from the nearest household.

1. Location B was in the middle of the village, approximately 100 m from the edge of the village, in an area with one human dwelling.
2. Location C was at the edge of the village, close to an area regularly cultivated for vegetables and rice using traditional irrigation systems.

To minimize positional bias on mosquito catches, the three MLBs were rotated nightly among the three locations to ensure that at the end of the 21 nights, each MLB had been at each of the three separate locations once every three nights. The order of the rotations of the three MLBs was randomised after every three nights to counter potential effects of any cyclical variations in the natural diurnal vector densities.

These experiments had no blank controls with no grids or without any bait, thus it was limited to comparing MLBs with varying surfaces area of grids. This was because our initial tests had demonstrated that un-baited MLBs did not attract host-seeking mosquitoes [12]. Furthermore, use of MLBs without grids as a control was not feasible since the MLB is designed as a lure-and-kill station without any trapping mechanism [12]. We therefore relied on previous published observations that mosquitoes visited baited-MLBs, but not un-baited ones and that these mosquitoes could be sampled intermittently during the night using drop nets [12]. Hence, no additional controls were necessary for the current study.

The experiments were done during the wet season (May-June 2013) and repeated in the dry season (August-September 2014), to capture the vector abundances at both times of the year.

### Semi-field evaluation to determine effects of odour-baited MLBs fitted with electrocuting grids on human-biting densities of female *An. arabiensis* mosquitoes

**Fig 3:**
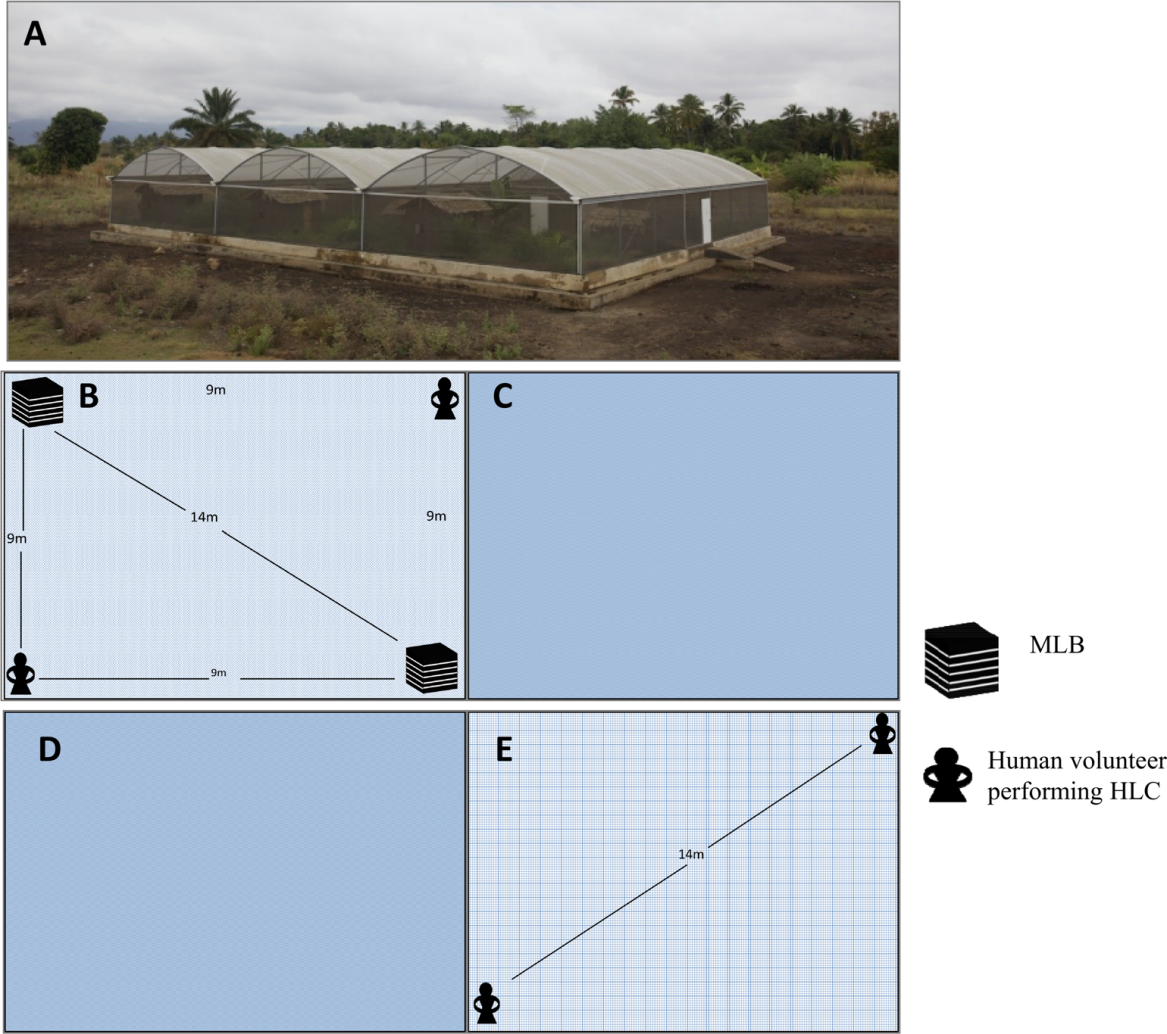
(A) A semi-field system (SFS) in the Kilombero district of south-eastern Tanzania, where semi-field study was carried out [40]. (B) Treatment chamber of the SFS contained odor- baited Mosquito Landing Box (MLB) and human volunteers performing human landing catching (HLC). (C and D) Empty chambers of the SFS, which were used as buffer to reduce inter-chamber bias during the orientation of host-seeking mosquitoes. (E) Control chamber of the SFS contained only volunteers performing HLC.

A comparative crossover study design was used in evaluating the effect of odour-baited MLBs fitted with electrocuting grids on human-biting densities against *An*. *arabiensis* for 12 nights (3 days/week) inside two equal sized chambers of a semi-field system (SFS) [40] (Fig 3A). Two SFS chambers were used whereby the treatment chamber contained two odour-baited MLBs fitted with three electrocuting grids each, and two human volunteers performing human landing catch (HLC) (Fig 3B). Each night, 500 nulliparous laboratory-reared unfed female *An*. *arabiensis* mosquitoes, aged 6–9 day old, were released at the centre of each SFS chamber two hours before the volunteers went in to begin HLC, so that the mosquitoes could acclimatise to the SFS environmental conditions. In the treatment chamber, the MLBs were placed at two diagonally opposite corners, 14 meters apart, on flat surfaces covered with clear plastic linoleum for easy visualization of any dead mosquitoes around the device (Fig 3B). The MLBs were baited with dirty nylon socks worn for 12 hours and carbon dioxide gas generated from yeast-molasses fermentation [44]. The MLBs were interchanged randomly inside the treated chamber nightly to minimize position bias. Pair of male human volunteers aged 18–30 years performing HLC were positioned at adjacent corners, so that they were also on diagonally opposite ends, 14 meters apart, and 9 meters from the adjacent MLB (Fig 3B). The HLC was carried out for 30 minutes at every two hours intervals from 2000Hrs through 0600Hrs, to collect mosquitoes landing and attempting to bite the volunteers. The remaining mosquitoes flying around in the chamber were collected using backpack aspirators from 0700Hrs to 0800Hrs. The two chambers of the SFS (Fig 3C and 3D) were left vacant acting as a buffer zone to minimize possible contamination between the treatment and control chambers. In the control chamber, a pair of male human volunteers aged 18–30 years, sat at two opposite corners performed HLC for 30 minutes at every two hours intervals, to collect mosquitoes landing and attempting to bite them (Fig 3E) in the absence of the MLB. Pairs of volunteers in each chamber were randomly interchanged nightly between treatment and control chambers to minimize bias due to differential attractiveness of individuals to mosquitoes [45,46].

### Mosquito collections, identification and assessment for *Plasmodium falciparum* infection

**Fig 4:**
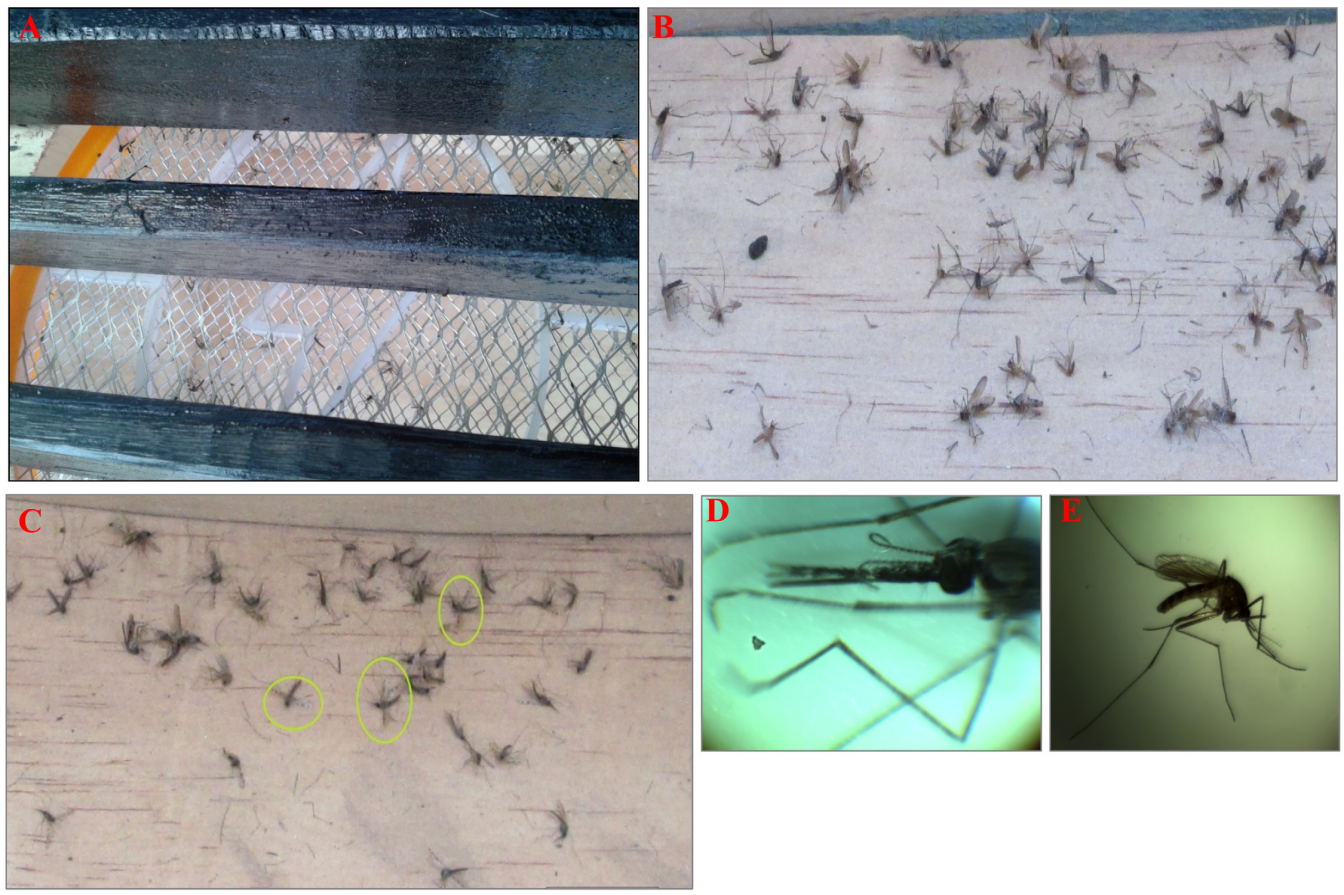
(A) Photograph of electrocuted wild mosquitoes stuck in the grid underneath the louvers of the Mosquito Landing Box (MLB). (B) A pile of mosquitoes after they fell from the electrocuting grid. (C) A close-up look of the collection of electrocuted *Anopheles gambiae s*.*l* (circled with spotted wings) and (D) A microscopic image of the head parts of the *Anopheles gambiae* s.l, and (E) all the morphologically features of the Culex species.

In the field experiments, mosquitoes were collected in the mornings. Electrocuted mosquitoes were gently removed individually from the EG surfaces using forceps (Fig 4), from the inside surfaces of the MLB and from the raised linoleum floors. A water moat was always placed at the base of the surfaces to prevent ants from scavenging on the mosquitoes. The mosquitoes were then identified morphologically and sorted by taxa and sex. Taxonomy was done using dichotomous keys for the *Anopheles* of Africa, south of the Sahara [47]. The mosquitoes were also sorted as either blood-fed or non-blood fed, gravid or semi-gravid.

Members of the *An*. *gambiae* complex and *An*. *funestus* group were stored separately for further individual species identification. A subsample of 690 *An*. *gambiae* s.l and 271 *An*. *funestus* group were randomly selected for species identification in the molecular laboratory at the Ifakara Health Institute using DNA-polymerase chain reaction (PCR) [48]. For the *An*. *gambiae* complex, multiplex PCR was done following the procedures of Scott *et al*. [49], while for the *An*. *funestus* group, DNA was extracted [50], and PCR was performed according to the procedures of Koekemoer *et al*. [51].

Female *Anopheles* mosquitoes identified as belonging to the *An*. *gambiae* complex and the *An*. *funestus* group were also separately pooled in groups of two (minimum) to ten (maximum) and stored for further examination to assess if they were infected with *P*. *falciparum* sporozoites, using circumsporozoite protein enzyme-linked immunosorbent assay (CSP-ELISA) techniques [52]. To remove false positives, all the sporozoite positive ELISA lysates were boiled at 100°C for 10 minutes to verify presence of *Plasmodium* protozoan antigen, which are heat stable [53,54].

For the semi-field experiments, each volunteer performing HLC recaptured only mosquitoes landing on him during scheduled collection periods of 30 minutes at every two hour intervals from 2000Hrs through 0600Hrs. Each morning, the leftover mosquitoes flying around in both chambers were collected using backpack aspirators. The number of mosquitoes killed by the MLB inside the treated chamber was recorded for the different MLBs after every two hours throughout the experimental night. All live collected mosquitoes were killed using petroleum vapours. By assessing the number of mosquitoes landing on the human volunteers inside the chambers with MLBs (treatment chamber) or without the MLBs (control chamber), we were able to assess whether presence of MLBs fitted with electrocuting grids in an enclosed environment affected the human-biting densities of laboratory-reared *An*. *arabiensis*.

## Data Analysis

Data were entered into excel spreadsheets and saved as comma-separated value (CSV) files. Data analysis was done using R statistical software version 3.1.1 [55]. Data were fitted using Generalized Linear Mixed Effects statistical Models (GLMMs) to describe effects of the different variables on mosquito catches. Since the data were evidently over-dispersed, we used the packages *lme4* [56], MASS and *glmmADMB* [57] to fit either Poisson distribution models or negative binomial distribution models with log-link functions to transform the data distributions. Numbers of individual mosquito species were assessed as a function of two fixed factors, the number of grids on the MLB and positions of the MLB, each time comparing additive versus multiplicative models, using Akaike Information Criteria (AIC) values [58]. Experimental days were treated as a random factor to account for the natural nightly heterogeneity in mosquito counts. The results of the parameter estimates were exponentiated to obtain the estimated mean number of mosquitoes killed each night by each MLB at different locations, and the associated relative rates (RR) of collecting a mosquito in any of the MLBs, when compared to the reference MLB with one grid, when located at the first location. The final data were summarised in tables and box plots to display the variability. Similarly, data collected from the semi-field studies on human biting densities between the control and treatment chambers where compared by using GLMMs, whereby number of mosquitoes recaptured was treated as a of function treatment, round and collection time. Number of mosquitoes recaptured in each chamber were treated as numeric variable, round and treatment as a factor variable.

## Results

### Molecular identification of the mosquito species

In all the experimental field tests in both dry and wet seasons, 9,685 mosquitoes in total were sampled from the three odour-baited mosquito landing boxes fitted with electrocuting grids. Of these, 3533 (36%) were members of the *Anopheles gambiae* complex, 107 (1%) were of *An*. *funestus* group, 826 (9%) were other *Anopheles* species, 1,230 (13%) were *Culex* species and 3989 (41%) were *Mansonia* species mosquitoes. Of the mosquitoes that were submitted for sibling species identification using PCR as described above, only 260 *An*. *gambiae s*.*l* and 57 from the *An*. *funestus* group were successfully identified. Of the *An*. *gambiae s*.*l*. samples, 98% (256/260) were *An*. *arabiensis*, while 2% (4/260) were *An*. *quadriannulatus*. For the *An*. *funestus* group, 54% (31/57) were identified as *An*. *rivulorum*, 25% (14/57) were *An*. *funestus s*.*s* and 21% (12/57) were *An*. *leesoni*. *An*. *arabiensis* and *An*. *funestus s*.*s* are the two predominant malaria-carrying vectors in the study area. We experienced high proportions of non-amplified mosquitoes during our molecular analysis for species identification, especially for the *An*. *funestus* group, (A summary of PCR results is presented in details in Table 1). Since *An*. *arabiensis* constituted 98% of the *An*. *gambiae* complex mosquitoes, the term *An*. *arabiensis* is used in subsequent sections to refer to all members of the complex.

**Table 1:** A summary of a sub-sample of the malaria vectors caught, that were submitted for sibling species identification using DNA-polymerase chain reaction (PCR) technique.

Species complex or group	Total number assayed by PCR	Total number and percentage successfully amplified	Percentage not amplified
***Anopheles gambiae* s.l**	690	Subtotal = 260: *Anopheles arabiensis*: 98% (256/260); *Anopheles quandianilatus*: 2% (4/260)	62% (430/690)
***Anopheles funestus* group**	271	Subtotal = 57: *Anopheles rivolurum*: 54% (31/57); *Anopheles funestus s*.*s*: 25% (14/57); *Anopheles lessoni*: 21% (12/57)	79% (214/271)
**Grand Total**	961	317	644

### Field tests conducted during the wet season

In total, 4,986 mosquitoes were collected from all three odour-baited MLBs equipped with EGs during the first round of the tests over the 21 nights in the wet season. Of these, there were 1,541 *An*. *arabiensis (*31%*)*, 38 *An*. *funestus* (1%), 356 mosquitoes belonging to any other *Anopheles* species (7%), 554 *Culex* species (11%), and 2,497 *Mansonia* species (50%). Ninety nine percentage of all the mosquitoes caught were non-blood fed, suggesting that they were host-seeking mosquitoes.

The estimated mean numbers of mosquitoes killed by each of the MLBs are shown in Tables 2 and 3. The number of mosquitoes collected increased with increasing grids in the MLBs. The MLB fitted with three grids killed significantly more *An*. *arabiensis* (RR = 1.59 [1.37–1.84], P < 0.001) than the MLB with just one grid, but significantly fewer of the other *Anopheles* species (RR = 0.41 [0.30–058], P < 0.001), and fewer *Mansonia* mosquitoes (0.73 [0.65–0.82], P < 0.001). The MLB with 3 grids also killed more *An*. *funestus* (RR = 1.56 [0.64–3.81], P = 0.335) and more *Culex* mosquitoes 1.90 [0.98–2.59], P = 0.329) although not by a statistically significant margin for either taxon. Similarly, the MLB fitted with two grids killed significantly more *An*. *arabiensis* (RR = 1.59 [1.37–1.84], P < 0.001) and more *Culex* mosquitoes (RR = 1.89 [1.03–2.64], P < 0.005) than the MLB fitted with one grid. However, the number of *An*. *funestus* killed by this MLB with two grids was similar to the MLB with one grid (RR = 0.95 [0.36–2.52], P = 0.913).

**Table 2:** Estimated mean number of *Anopheles* mosquitoes collected per night from the odour-baited mosquito landing boxes (MLBs) fitted with one, two or three electrocuting grids during the wet season or dry season tests^ϕ^.

		***Anopheles arabiensis***			***Anopheles funestus***			**Other *Anopheles***		
**Round 1 (Wet season)**	**No. Grids on MLB**	Mean [95% CI]	RR [95% CI]	P value	Mean [95% CI]	RR [95% CI]	P value	Mean [95% CI]	RR [95% CI]	P value
	One Grid	13.82 [9.90–19.28]	REF	REF	0.15 [0.05–0.45]	REF	REF	1.51 [0.73–3.13]	REF	REF
	Two Grids	21.97 [18.95–25.47]	1.59 [1.37–1.84]	<0.001	0.14 [0.05–0.36]	0.95 [0.36–2.52]	0.913	1.14 [0.12–10.71]	0.75 [0.08–7.09]	0.049
	Three Grids	36.04 [9.16–141.76]	2.61 [0.66–10.26]	<0.001	0.23 [0.09–0.55]	1.56 [0.64–3.81]	0.335	0.63 [0.45–0.88]	0.41 [0.30–0.58]	<0.001
		***Anopheles arabiensis***			***Anopheles funestus***			**Other *Anopheles***		
**Round 2 (Dry season)**	**No. Grids on MLB**	Mean [95% CI]	RR [95% CI]	P value	Mean [95% CI]	RR [95% CI]	P value	Mean [95% CI]	RR [95% CI]	P value
	One Grid	17.67 [15.64–19.96]	REF	REF	0.03 [0.01–0.14]	REF	REF	0.21 [0.09–0.47]	REF	REF
	Two Grids	32.65 [29.07–36.67]	1.85 [1.65–2.08]	<0.001	0.13 [0.06–0.31]	4.79 [2.08–11.02]	<0.001	0.49 [0.32–0.73]	2.35 [1.56–3.53]	<0.001
	Three Grids	28.79 [25.54–32.45]	1.63 [1.45–1.84]	<0.001	0.02 [0.01–0.05]	0.73 [0.29–1.85]	0.515	0.40 [0.26–0.63]	1.95 [1.25–3.04]	0.003

ϕ The RR stands for relative rate while REF stands for a reference category.

All the estimations were generated using Generalized Linear Mixed Effects Models in R [55].

**Table 3:** Estimated mean number of non-malaria mosquitoes and overall number of mosquitoes collected per night from the odour-baited mosquito landing boxes (MLBs) fitted with one, two or three electrocuting grids during the wet season or dry season tests^ϕ^.

		***Culex* species**			***Mansonia* species**			**All mosquito species combined**		
**Round 1 (Wet season)**	**No. Grids on MLB**	Mean [95% CI]	RR [95% CI]	P value	Mean [95% CI]	RR [95% CI]	P value	Mean [95% CI]	RR [95% CI]	P value
	One Grid	2.61 [1.79–3.81]	REF	REF	14.66 [10.87–19.75]	REF	REF	20.24 [14.72–27.84]	REF	REF
	Two Grids	4.39 [2.68–4.88]	1.89 [1.03–2.64]	0.029	12.08 [10.85–13.45]	0.82 [0.74–0.92	<0.001	99.36 [82.35–119.89]	4.91 [4.07–5.92]	<0.001
	Three Grids	3.39 [2.56–4.15]	1.90 [0.98–2.59]	0.329	10.68 [9.50–12.01]	0.73 [0.65–0.82]	<0.001	41.53 [34.37–50.19]	2.05 [1.69–2.48]	<0.001
		***Culex* species**			***Mansonia* species**			**All mosquito species combined**		
**Round 2 (Dry season)**	**No. Grids on MLB**	Mean [95% CI]	RR [95% CI]	P value	Mean [95% CI]	RR [95% CI]	P value	Mean [95% CI]	RR [95% CI]	P value
	One Grid	1.93 [1.24–2.99]	REF	REF	1.59 [1.01–2.51]	REF	REF	19.07 [13.77–26.41	REF	REF
	Two Grids	2.78 [2.24–3.44]	1.44 [1.16–1.79]	<0.001	2.05 [1.65–2.54	1.29 [1.04–1.59]	0.02	30.02 [27.77–32.45]	1.57 [1.46–1.70	<0.001
	Three Grids	3.70 [2.97–4.62]	1.92 [1.54–2.39]	<0.001	4.16 [3.32–5.21]	2.61 [2.08–3.27]	<0.001	33.05 [30.49–35.82]	1.73 [1.59–1.88]	<0.001

ϕ The RR stands for relative rate while REF stands for a reference category.

All the estimations were generated using Generalized Linear Mixed Effects Models in R [55].

We also observed statistically significant effects of location on number of mosquitoes of different species collected. When any of the MLBs, regardless of number of grids, was at position C at the edge of the village close to an area regularly cultivated for vegetables and rice using traditional irrigation systems, we collected at least 4 times more mosquitoes of all species combined than at position A inside the village, approximately 150 m from the edge of the village. Specifically, at position C, we collected 5.4 times more *An*. *funestus* (RR = 5.37 [2.04–14.15], P < 0.001), 5.3 times more of the other *Anopheles* mosquitoes (RR = 5.26 [3.83–7.23], P < 0.001), 4.2 times more *Culex* (RR = 4.23 [3.28–5.46], P < 0.001) and 5.4 times more *Mansonia* mosquitoes (RR = 5.43 [4.79–6.14], P < 0.001) than at position A. However, there were significantly fewer *An*. *arabiensis* mosquitoes sampled when the MLB was at position C relative to position A (RR = 0.70 [0.62–0.80], P < 0.001). Similarly, we collected more of the non-malaria mosquitoes, *Culex* (RR = 1.68 [1.26–2.24], P < 0.001), other *Anopheles* species (RR = 1.18 [0.81–1.73], P = 0.39) and *Mansonia* (RR = 1.63 [1.41–1.88), P < 0.001)) at position B in the middle of the village (approximately 100 m from the edge of the village, in an area with one human dwelling near it), but fewer *An*. *arabiensis* (RR = 0.83 [0.72–0.94, P = 0.01) and the same number of *An*. *funestus* (RR = 0.99 [0.29–3.46], P = 0.99) compared to position A.

### Field tests conducted during the dry season

Results were generally similar to those of the wet season and are given in Tables 2 and 3. Overall, 4,699 mosquitoes were collected from all the three odour-baited MLBs fitted with electric grids during the dry season tests. Of these, 1,992 (42%) were *An*. *arabiensis*, 69 (2%) were *An*. *funestus*, 470 (10%) were other *Anopheles* species, 676 (14%) were *Culex* species and 1,492 (32%) were *Mansonia* species. Relative to the MLB with one grid, significantly more mosquitoes (all species combined) were collected at the MLB with three grids (RR = 1.73 [1.59–1.88], P < 0.001) or the one with two grids (RR = 1.57 [1.46–1.70], P < 0.001). Regarding effect of position, we also observed that relative to position A, 3.8 times more mosquitoes of all species were collected when MLBs were at position C (RR = 3.84 [3.53–4.17], P < 0.001), and 1.8 times more when MLBs were at position B (RR = 1.83 [1.68–2.00], P < 0.001).

Upon morphological identification, we determined that the MLB with three grids killed significantly more *An*. *arabiensis*, (RR = 1.63 [1.45–1.84], P < 0.001), more *Culex* mosquitoes (RR = 1.92 [1.54–2.39], P < 0.001) and more *Mansonia* mosquitoes (RR = 4.16 [3.32–5.21], P < 0.001) than the MLB with one grid. There was no significant difference in numbers of *An*. *funestus* caught from the MLB with three grids compared to the MLB with one grid. The MLB with two grids also killed more *An*. *arabiensis* (RR = 1.85 [1.65–2.08], P < 0.001), more *An*. *funestus* (RR = 4.79 [2.08–11.02], P < 0.001) more *Culex* (RR = 1.44 [1.16–1.79], P < 0.001) and more *Mansonia* mosquitoes (RR = 1.29 [1.04–1.59], P < 0.02), compared to the MLB with one grid.

**Fig 5:**
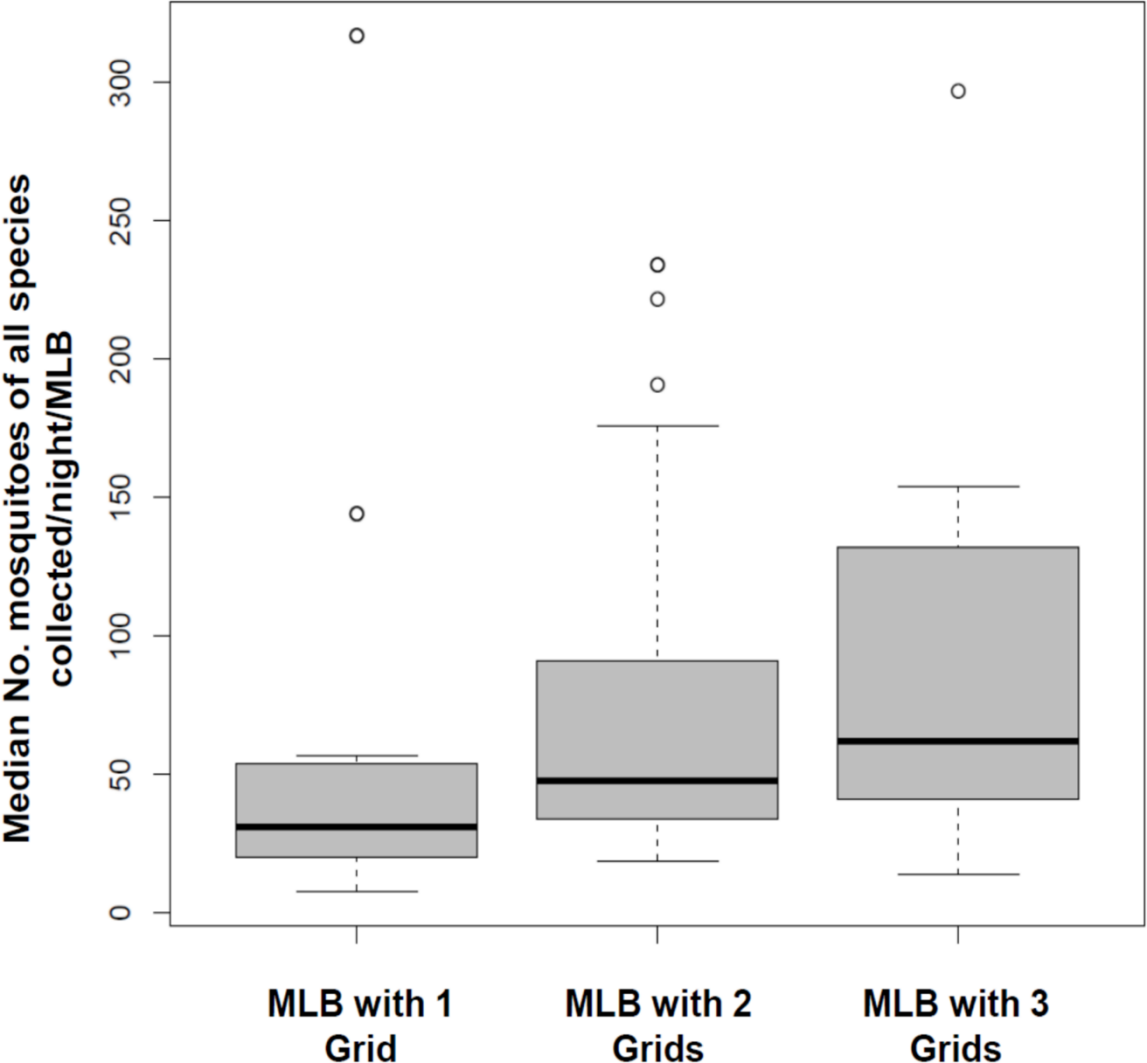
Comparison of mosquitoes (all species combined) killed by odour-baited mosquito landing boxes fitted with one, two or three electrocuting grids on the sides.

**Fig 6:**
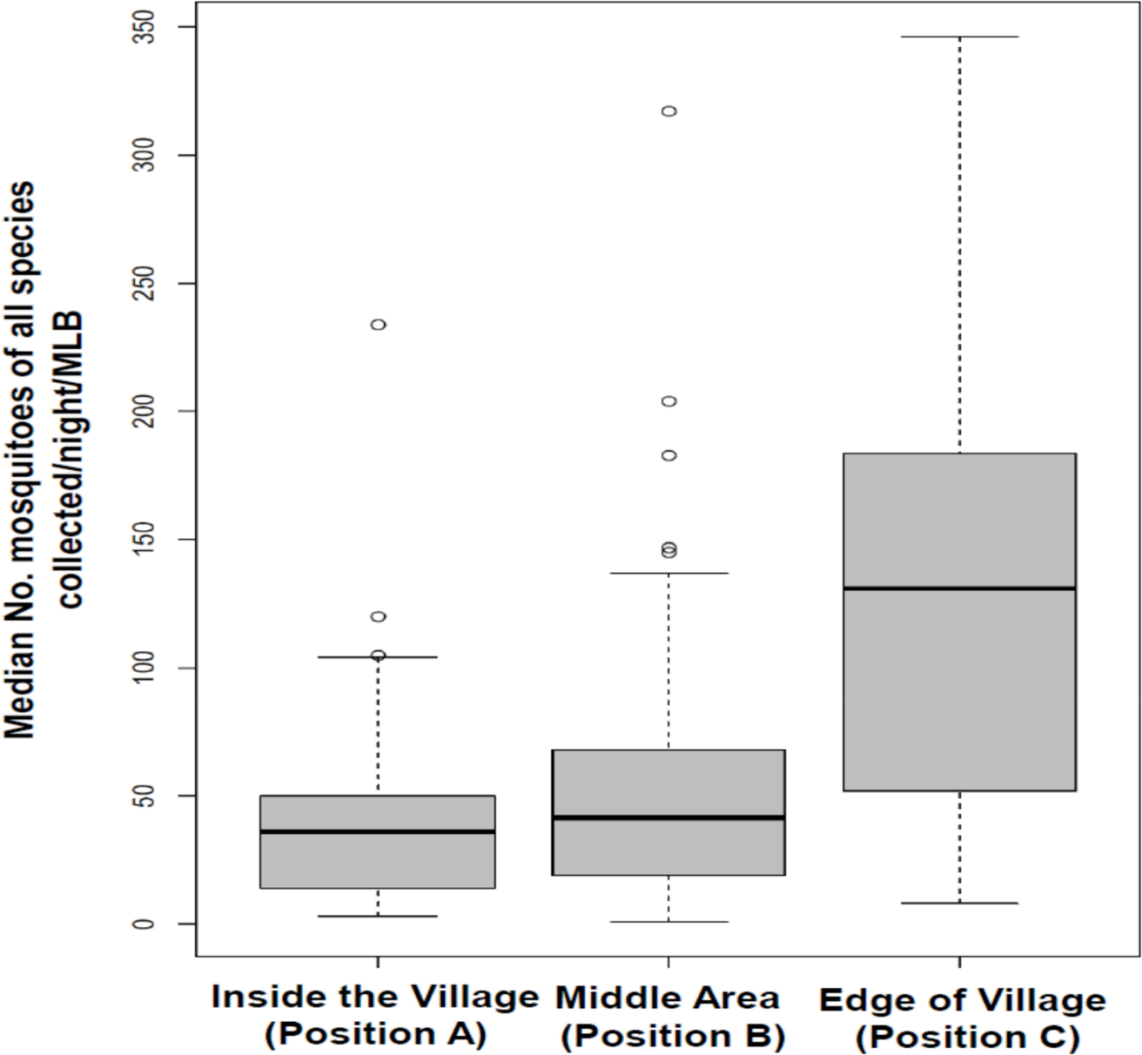
Comparison of mosquitoes (all species combined) that were collected when the odour-baited mosquito landing boxes were located at the three different positions.

Interestingly, there was no difference in the number of mosquitoes collected between the wet season (first round) and the dry season (second round) except for *Mansonia* species, for which there were higher numbers in the wet season relative to the dry season (RR = 1.80 [1.17–2.79], P < 0.007). Figs 5 and 6 show the variations in mosquito collections by number of grids and positions respectively.

### *Plasmodium* infection rates in the field-sampled mosquitoes

In total, 1,230 individual mosquitoes from the *An*. *gambiae* complex and the *An*. *funestus* group were tested for *P*. *falciparum* circumsporozoite protein (CSP). We used a modified enzyme-linked immunosorbent assay (ELISA) technique that involved boiling the ELISA lysate for 10 minutes at 100°C, to exclude any heat stable antigens that may occasionally express as false positives in CSP-ELISAs [53], which is highly likely in zoophilic *Anopheles* species [54,59]. The initial sporozoite rate for *An*. *arabiensis* was 0.84% (i.e. 8 out of 958) but after boiling, the rate dropped to 0.2% (2 of 958). None of the *An*. *funestus* (in which half were *An*. *rivulorum*) tested positive for *P*. *falciparum* circumsporozoite protein.

### Comparisons of number of *An*. *arabiensis* mosquitoes landing on volunteers inside semi-field chambers with or without MLBs fitted with electrocuting grids

**Fig 7:**
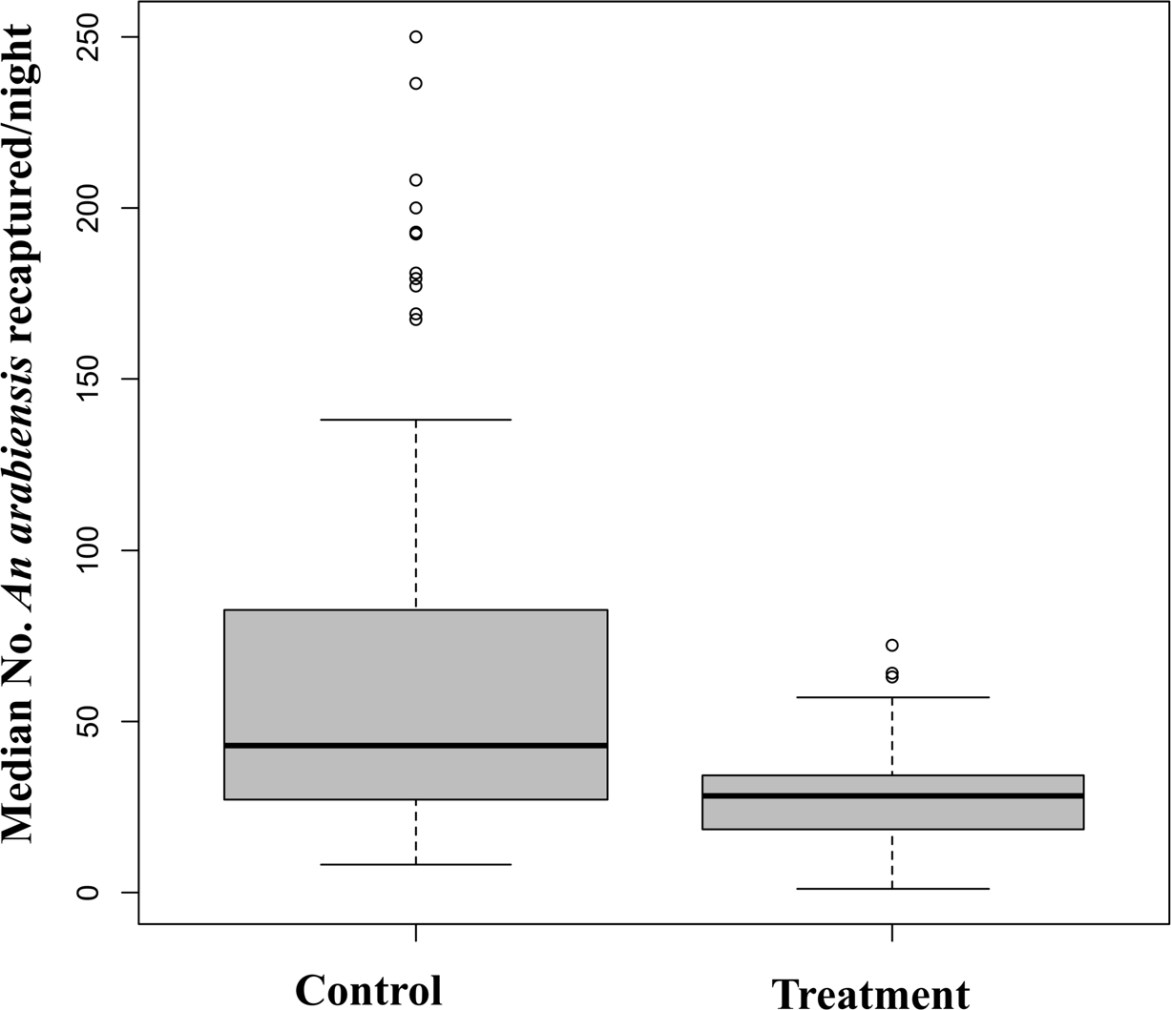
Mean number of live mosquitoes recaptured for 30 minutes collection sessions at each 2 hours intervals by human landing catch (HLC) in the control and treatment chambers.

Overall, there were statistically more *An*. *arabiensis* mosquitoes collected by HLC inside the control chambers without MLBs than in the treatment chambers with MLBs (P < 0.001). Despite the daily replenishment of the adult mosquito populations, there were still 57.2% fewer mosquitoes in the treatment chamber than in the controls, presumably because they were killed by the MLBs. Of the total of 6,000 female *An*. *arabiensis* mosquitoes released in control chamber at a rate of 500/night over the 12 nights of testing, the two volunteers recaptured a total of 2,791 (46.6%) and 2,866 (47.8%), respectively. On the other hand, of the 6,000 female *An*. *arabiensis* mosquitoes released over the 12 nights of testing in treatment chambers, where 2 MLBs fitted with electrocuting grids had been located, the two volunteers recaptured only 1,194 (19.9%) and 1,229 (20.5%), respectively. When the mean numbers of live mosquitoes recaptured in each chamber at each interval of collection were compared, the result showed significantly higher numbers of live mosquitoes recaptured in the control than in the treated chamber (Fig 7). Each night, four times fewer mosquitoes (RR = 4.39 [3.92–4.93], P <0.001), were recaptured alive from the treatment chamber with MLB, which indicated the killing effect of the device fitted with electrocuting grids inside the treated chamber against the control setup. The mean nightly number of mosquitoes caught in the chamber with MLB was 14.27 [10.77–18.89], while the mean in the chamber without MLB was 62.69 [53.06–74.07].

**Fig 8:**
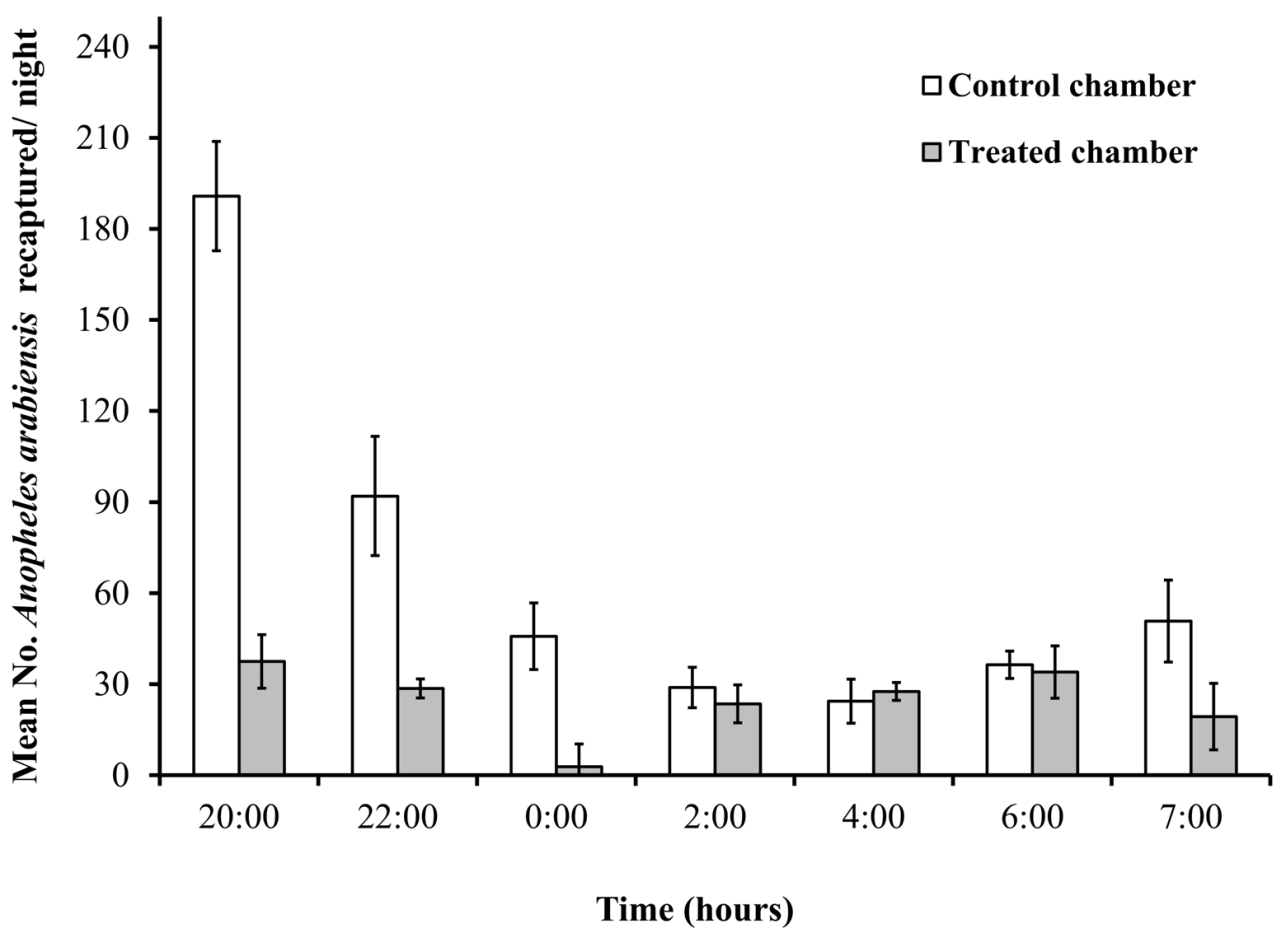
Mean number of live adult *An*. *arabiensis* mosquitoes recaptured by human landing catch (HLC) during each of the half-hour collection sessions at the end of each 2-hour interval in the treatment chamber with the MLBs and in the control chamber without MLBs.

Since the collections were conducted after every two hours intervals, we could assess the times of night when the host-seeking activity was strongest, and therefore when most of the released mosquitoes were recaptured. Most of the recaptured mosquitoes had been collected at the beginning of night from 2000Hrs to 2200Hrs, and the number gradually decreased with time in both the control and treatment chambers, though there was a minor increase in the morning collection at 0700Hrs (Fig 8).

## Discussion

In Africa, malaria transmission still occurs primarily indoors [60,61], but the relative proportion that occurs outdoors is increasing, particularly in communities where indoor interventions are used extensively [6,62]. Odour-baited technologies for attracting and killing disease vectors by using lethal insecticide targets have been considered as potential options against outdoor-biting malaria vectors [11]. Such technologies must be designed to avoid known vector control challenges such as development of insecticide resistance after prolonged use as well as behavioural resilience or avoidance by some vector species or populations [20,63].

The Mosquito Landing Box (MLB) [12], fitted with electrocuting grids (EGs) may offer a quick-acting, non-chemical control mechanism against outdoor-biting malaria vectors, even if these vectors spend only brief periods on the device, and even if the mosquitoes are physiologically resistant to insecticides commonly used for vector control. Furthermore, grids may reduce associated labour costs and eliminate the need for insecticides, thus improving environmental safety and create opportunities to address the management of insecticide resistance [64] in mosquito populations. The addition of a light-based sensor ensured that the device is automatically switched on at dusk and off at dawn also conserves energy, increases longevity of the odour lure as it was being emanated only at night, and eliminates unwanted effects on daytime flying non-target insects. This also improves the safety associated with the device, especially in places where children might play and touch the devices during the day.

The number of mosquitoes collected increased with increased number of grids in the MLB. Significantly more mosquitoes of all species were killed by the three-grid MLB relative to one-grid MLB, and slightly more by the two-grid MLB than the one-grid MLB, a trend attributable to the increase in mosquito contact surface areas when the number of grids is increased. *An*. *arabiensis* was the most abundant member of the *An*. *gambiae* complex identified representing 98% of the total sample size. Only one other member of the complex, *An*. *quadriannulatus*, was found. We also have extensive evidence from previous indoor and outdoor studies that *An*. *gambiae s*.*s*., which was formerly the dominant sibling species in this complex, is now nearly extinct [4]. The MLB prototype that we used is designed specifically to target outdoor-biting vector populations, regardless of species. Moreover, its effects on non-malaria mosquitoes such as *Culex* and *Mansonia* species, as we observed in this study could improve its level of acceptability, as it would also be effective against other mosquito-borne illnesses and against nuisance biting mosquitoes.

The majority of the other *Anopheles* mosquitoes caught during the experiments were morphologically identified as *An*. *coustani* (considered as secondary malaria vectors). Since vector species such as *An*. *arabiensis* seek blood hosts both outdoors and indoors, depending on availability of the hosts [7,8], efficacy of the MLB against this species suggests that the device can be effective as a complementary and non-chemical option targeting them. Interestingly, some of the *An*. *funestus s*.*s*., that are known to feed and rest mainly indoors, were caught outdoors. However, it is unknown what proportion of the population the outdoor collections represent, as indoor collections were not conducted as part of this study. Since *An*. *funestus* is known to be a major contributor to the on-going residual malaria transmission in east Africa [65,66] as well as a recent study in west Africa by Moiroux *et al*. [62,67]. This observation further justifies the need for supplementary interventions to target these species that are increasingly biting outdoors.

The ELISA assays determined that the sprozoite rate was 0.84% for *An*. *arabiensis* before boiling, but only 0.2% was confirmed positive after boiling. False positive results are known to occur with samples that feed on animals, such as *An*. *parensis* and the *An*. *marshallii* group [53,54,59]. None of the *An*. *funestus*, of which half were *An*. *rivulorum*, were found positive with malaria parasites. However, further collections and analyses should be done since this species previously has been found infectious with malaria sporozoites [68–70], particularly in areas with widespread indoor interventions [70]. No ELISA was done on other *Anopheles* species, the majority of which were *An*. *coustani*. However, the earlier work done by Gillies & De Meillon [71] in Tanzania, following the dissection of salivary gland, 1 out of the 567 dissected *An*. *coustani* was found positive with malaria parasites, and yet there was no evidence that this species had a minor potential malaria transmission. Recently, there has been a suggestion that this species may be a secondary vectors that might play a role in malaria transmission in the Taveta area of Kenya [72], however the ELISA tests done in that study were not confirmed by either boiling the lysate or doing PCR and the results must therefore be questioned. This species has also been reported to be a vector of the Rift Valley fever virus during the disease epidemics [73]. Although the main target of this study was malaria mosquitoes (for which the estimated mean catches are shown in (Table 2), it also was important to assess overall impact of the devices on all mosquito species combined, including the culicines. This is particularly important as the biting densities of non-malaria mosquitoes could influence people’s perception on whether a malaria control intervention is effective [74].

The differences due to location are important when determining optimal positions necessary to achieve maximum impact on vector densities. For example, at least four times more mosquitoes of all species were collected when the MLB (regardless of number of grids) was placed at position C, which was at the edge of the village, nearest to vector breeding sites, relative to the device at position A, which was 150 meters from the nearest mosquito breeding site and inside the village (Fig 6). The present results match the findings of many previous reports on strong associations between high numbers of adult mosquito densities and distance from nearest aquatic breeding sites [75,76]. Similar observations have also been made of relationships between increases in malaria cases in houses near mosquito breeding sites, suggesting that general mosquito control interventions should be either more focused on the households close to the breeding sites or located to intercept mosquitoes flying between these aquatic sites and human dwellings [77,78].

Unexpectedly, there was no difference in number of mosquito catches at the end of the wet season relative to the dry season, except for *Mansonia* species that increased significantly at the end of the wet season, perhaps because the study area is a rice growing area with breeding sites available throughout the year [79,80]. Moreover, some species, such as *An*. *arabiensis*, tend to maintain their population size throughout most of the year even when the breeding sites are dry, since their eggs can remain dormant to resist desiccation [81]. Also, the persistent number of *An*. *funestus* during the dry season may be due to their preference for breeding in permanent water bodies and as well as in hidden, small, man-made habitats [82].

The mosquito lure used here has been demonstrated previously to attract be more proportions of mosquito species than humans at long-range [42]. During these experiments, the synthetic lure used [42], was prepared by hand and dispensed using locally available nylon strips [43]. However, the blend have been lately improved and packed into simple long-lasting pellets that can be widely used hence reducing cost and time for labour. Similar techniques are already being implemented by commercial manufacturers such as Biogents (BG) Ltd (Germany) for large-scale use against the dengue vector *Ae*. *aegypti* [83]. No other insects (non-target organisms) were captured suggesting that the MLB baited with the synthetic lures that we used here selectively attract mosquitoes and have minimal environmental impact. Moreover, in this study, nearly all female mosquitoes collected at any of the positions were not blood fed, suggesting that they were in a host-seeking state or looking for a resting site after emerging. Additional studies should be done to determine whether a non-odour-baited MLB could also acts as an attractive outdoor resting box. As an effective control tool against such vector populations, the MLB would be effective against outdoor-biting vectors, which otherwise perpetuate residual malaria, even where indoor interventions already are common.

In the semi-field assays that we conducted to assess potential effects of the electrocuting grid-fitted MLBs on human-biting densities of the vectors, the results clearly demonstrate that at medium range of host seeking (10 metres) MLBs were significantly more attractive to mosquitoes than the human volunteers and reduced human biting densities. The reduction in mosquitoes recaptured by HLC from the treatment chamber compared to the control chamber showed the impact of MLB fitted with the electrocuting grids in removing host seeking vectors from the environment. In this experiment, we also observed that more mosquitoes were recaptured at the beginning of experimental nights in both control and treatment chambers than later in the night, although there was a small peak also in the morning (Fig 8). This pattern matches the naturally observed nightly biting patterns of *An*. *arabiensis* in many parts of Africa [5] and such high numbers of mosquitoes recaptured during the early hours of the night have been reported previously [25,84]. While this experiment did not allow us to assess the performance of the device at varying vector abundances or in the presence of LLIN use, the apparent mass killing effect demonstrated in the study suggests we can achieve desirable levels of community protection, if the device is used consistently including during dry seasons when densities are low. When the device is scaled up, both temporal and spatial targeting will be essential to ensure maximum benefit.

We have demonstrated that the improved Mosquito Landing Boxes with electrocuting grids are effective weapons against malaria vectors and non-malaria vectors outdoors. The MLB might also be used as a surveillance tool to measure vector population densities and disease transmission outdoors. Since the MLB is baited with synthetic human lures, it is an alternative to the ethically questionable practice of human landing catches to potentially infectious mosquitoes. The HLC method subjects volunteers to an increased risk of being bitten by infectious mosquitoes [85] and is extremely arduous and labour intensive. Also, due to variability between individuals’ attractiveness to mosquitoes and ability to catch landing mosquitoes, having a standardised system that measures outdoor man-vector contact in a similar way across and between sites, as is done with the CDC light trap for indoor biting mosquitoes [86], is highly desirable.

In addition to attracting and killing potentially infectious and nuisance mosquitoes, the MLB may be able to supply lighting to households in rural communities from the power from its solar panel. This concept was originally aimed at reducing potential barriers to acceptance of the MLB by community members and may significantly increase uptake because users have an obvious personal benefit to use the device. The “home lighting” concept has been demonstrated on a small scale in rural south-eastern Tanzanian communities (Okumu et al., unpublished data), where the main electricity coverage remains below 10% [87–89]. In those demonstrations, nine specially designed experimental huts and two village houses, were supplied with solar-powered lighting systems, using the same solar panel that powers the odour-dispensing unit and electrocuting grids in the MLBs. The MLBs could improve livelihoods by controlling disease vectors and providing basic lighting, thus reducing risks associated with common rural light sources such as kerosene lamps. We expect that since the MLBs can provide energy for basic lighting, pupils’ home-study, and mobile phone charging, they stand to improve opportunities for acceptability and sustainability of this technology in rural and remote communities. However, future social-anthropological studies are recommended to assess the community acceptability on the cost, maintenance and sustainability of the MLB.

One limitation of this device is the need for CO_2_ gas, a major component of the mosquito lures that we used. In the field studies we used industrial CO_2_ gas, delivered in pressurized cylinders and dispensed through calibrated flow metres (Glass Precision Engineering Ltd., United Kingdom). The CO_2_ gas is an important component of mosquito lures. It is necessary for activating mosquitoes and synergizing other lure components [90,91]. While industrial CO_2_ is effective for experimental and demonstration purposes, it is unsustainable on a large-scale in rural sub-Saharan Africa, but there are several alternatives including CO_2_ gas generated from yeast-sugar fermentation [92] or yeast-molasses fermentation [44], as we used in our semi-field tests. For field applications, we can consider either alternative or host odours suctioned directly from human dwellings [12,93,94].

Another limitation of our study was the high proportion of non-amplified field mosquitoes during our molecular analysis for species identification. We could not determine causes of the poor amplification rates in this study, which was also observed in other studies ongoing at the same time. However, we hypothesised problems arising from suboptimal specimen handling, preservation and storage conditions, and long waiting times for analysis that that degrade the DNA. Also in our laboratory, we have been using single legs or wing only during DNA extraction processes that might produce less DNA than extraction from the abdomen or legs and wings. Also, it might be a technical problem in the laboratory as similar challenge has been experienced by other scientists who have been using the same laboratory during the same period. Some of the non-amplified samples were re-assayed in a separate laboratory and confirmed successfully amplified. We therefore have also a similar plan to repeat these assays on the non-amplified samples in a separate laboratory.

## Conclusion and Recommendations

This study has shown that it is possible to target outdoor-biting mosquitoes, including major malaria vectors, using the odour-baited mosquito landing box (MLB) fitted with affordable electrocuting grids and automated sensors. It also demonstrated that using MLBs close to humans reduced their risk of mosquitoes attempting to feed on them sowing that the odour blend is effective at medium range and is not likely to lure in mosquitoes that then go and bite people located close to the traps, for instance if used in the peri-domestic space. This non-chemical technology instantly kills host-seeking mosquitoes that would naturally bite outdoors or those that might have changed behaviours from indoor-biting and now increasingly bite outdoors due to selection pressure from the indoor insecticide-based interventions such as LLINS and IRS. In addition, this method may be effective in managing vector populations that are physiologically resistant to insecticide-based interventions, as was observed in the field studies reported here, conducted in an area where malaria vectors are resistant to pyrethroids. Electric grids eliminate the need for applying insecticides to the MLB, and ensure high killing efficacy even if the mosquitoes are behaviourally or physiologically resistant. The MLB may allow us to preserve the effectiveness of the existing interventions such as LLINs and IRS by removing resistance alleles from the population using an entirely new insecticide-free killing strategy. Moreover, MLBs with electrocuting grids might support an integrated approach for controlling and for effectively monitoring of densities and infectiousness of disease-transmitting mosquitoes without the need for human landing catches, the current gold standard method for measuring outdoor mosquito densities. Other than the malaria vectors, the MLB could target simultaneously non-malaria mosquitoes such as *Culex* and *Mansonia* species that are potential vectors of lymphatic filariasis and arboviruses, and are also major sources of biting nuisance to humans. Reducing nuisance biting will increase consumer satisfaction with this method. Further studies are recommended to assess and compare the monitoring efficacy of the MLB equipped with grids with other existing tools, as well as the potential effects of the tool on malaria transmission and incidence rates in communities. We also suggest comparative evaluation studies using MLBs with grids to determine species variations on outdoor collections relative to indoor mosquito populations.

## Ethical Consideration

Prior to the commencement of the study, a detailed explanation of the aims, study procedures, risks and benefits were provided to the owners of the land (Position A and B) where the MLBs were located. Position C is a hired area by Ifakara Health Institute (IHI) where experimental huts are located near the irrigation rice fields for field trials. Written consents were sought from the head of the households upon agreement to be involved in the study. All the households that were located nearby the study areas were provided with bed nets and topical repellents. Participation was voluntary and household members were free to withdraw from the study in case of any inconveniences. However, none of the study participants withdrew from the study.

For semi-field experiments, both verbal and written consents forms were sought from the volunteers prior to the start of the experiment. To reduce the risks of being bitten by mosquitoes, the volunteers were provided with long sleeved jackets with ventilated hoods and gloves during mosquito collections. The volunteers received free access to malaria tests using Rapid Diagnostic Test (RDT) and they had access to malaria treatment using the first line drug, artemether lumefantrine (Coartem), in case of any malaria cases (which in this particular experiment there was none). We did not provide chemoprophylaxis to the volunteers because mosquitoes used were laboratory-reared females with no prior blood-meals.

Ethical review and approval was granted by the institutional review board of Ifakara Health Institute (Ref: IHI/IRB/NO.030) and The Medical Research Coordinating Committee at the National Institute of Medical Research in Tanzania (Ref: NIMR/HQ/R.8a/Vol.IX/1222). An ethics waiver was provided from the Human Research Ethics Committee of the University of the Witwatersrand, Johannesburg. The permission to publish this study was granted by the director general of the National Institute of Medical Research in Tanzania (Ref: NIMR/HQ/P.12 VOL XVII/19).
